# Evaluation of a learner-designed course for teaching health research skills in Ghana

**DOI:** 10.1186/1472-6920-7-18

**Published:** 2007-06-27

**Authors:** Imelda Bates, Daniel Ansong, George Bedu-Addo, Tsiri Agbenyega, Alex Yaw Osei Akoto, Anthony Nsiah-Asare, Patrick Karikari

**Affiliations:** 1 Disease Control Strategy Group, Liverpool School of Tropical Medicine, UK; 2 Department of Child Health, Komfo Anokye Teaching Hospital, Ghana; 3 Department of Medicine, Komfo Anokye Teaching Hospital, Ghana; 4 Dean, School of Medical Sciences, Kwame Nkrumah University of Science and Technology, Ghana; 5 Chief Executive Officer, Komfo Anokye Teaching Hospital, Ghana; 6 Medical Director, Komfo Anokye Teaching Hospital, Ghana

## Abstract

**Background:**

In developing countries the ability to conduct locally-relevant health research and high quality education are key tools in the fight against poverty. The objective of our study was to evaluate the effectiveness of a novel UK accredited, learner-designed research skills course delivered in a teaching hospital in Ghana.

**Methods:**

Study participants were 15 mixed speciality health professionals from Komfo Anokye Teaching Hospital, Kumasi, Ghana. Effectiveness measures included process, content and outcome indicators to evaluate changes in learners' confidence and competence in research, and assessment of the impact of the course on changing research-related thinking and behaviour. Results were verified using two independent methods.

**Results:**

14/15 learners gained research competence assessed against UK Quality Assurance Agency criteria. After the course there was a 36% increase in the groups' positive responses to statements concerning confidence in research-related attitudes, intentions and actions. The greatest improvement (45% increase) was in learners' actions, which focused on strengthening institutional research capacity. 79% of paired before/after responses indicated positive changes in individual learners' research-related attitudes (n = 53), 81% in intention (n = 52) and 85% in action (n = 52). The course had increased learners' confidence to start and manage research, and enhanced life-long skills such as reflective practice and self-confidence. Doing their own research within the work environment, reflecting on personal research experiences and utilising peer support and pooled knowledge were critical elements that promoted learning.

**Conclusion:**

Learners in Ghana were able to design and undertake a novel course that developed individual and institutional research capacity and met international standards. Learning by doing and a supportive peer community at work were critical elements in promoting learning in this environment where tutors were scarce. Our study provides a model for delivering and evaluating innovative educational interventions in developing countries to assess whether they meet external quality criteria and achieve their objectives.

## Background

Access to high quality education has been recognised as a key tool in the fight against poverty in developing countries [1]. The UK's International Education Strategy complements these international priorities by promoting quality assured outlets for education, supporting worldwide improvement of education, particularly in Africa, and developing UK universities as international hubs for learning and research [2]. To fulfil these national and international education goals in promoting research skills overseas, UK universities need to demonstrate that they can provide programmes that meet local and overseas research needs while maintaining educational quality standards [3].

Komfo Anokye Teaching Hospital (KATH) in Kumasi, Ghana aims to become a centre of teaching and research excellence within west Africa. As part of a programme to increase the institutional capacity of KATH to support and conduct research and to use research results to improve patient care we have developed a one-year part-time course to equip health professionals in KATH with basic research skills. Educational theories, emanating predominantly from developed countries, such as those concerning social learning [4] and learning at work [5] informed the design of this course. Social learning theories suggest that learning can be promoted through 'communities of practice' in which members (in this case, students at KATH) are mutually engaged on a joint enterprise (in this case, enrolled on a research course) and undertake a shared repertoire of actions (in this case, research-related activities) [6]. Theories related to learning at work highlight the synergy between the research skills being taught and the learners' environment at work so the students were encouraged to engage departmental colleagues in identifying priority clinical problems for research, in assisting with the design and implementation of the research, and in utilising research results to improve patient care. Through this approach, and because students remained at post throughout the course, their learning would be encouraged and reinforced by colleagues within the work environment [7,8].

The planned course outcome was *learners who were competent and confident in basic research skills*. To successfully complete the course learners had to devise, conduct and write up their own piece of research. The course consisted of two one-week workshops (in months one and eight), monthly peer group meetings, seminars on biostatistics, data analysis and internet literature searches, and meetings with departmental heads and supervisors. The workshops were facilitated by UK and in-country tutors using methods such as group work, short talks, demonstrations, and self-directed learning. Peer group meetings focused on specific academic and logistics issues, and helped students keep to their time plans. The course assessments contributed to the learning process [9,10] and consisted of a research proposal, a project report and a written reflection about skills that learners had acquired through doing research. Students had to satisfactorily complete all three assignments to pass the course.

The learning outcomes, course curriculum, timetable, content, assessments and marking criteria were devised by the first cohort of learners in an iterative, reflective process guided by UK facilitators. During these facilitated sessions the learners identified the skills they should acquire to be able to design and manage a simple research project. They used this list of skills to write learning outcomes for the course and to devise assessments that would demonstrate that the learning outcomes had been met. They also drafted marking schemes for their assignments and agreed deadlines for handing in assignments.

To complete the course successfully the students needed to engage with a wide group of work colleagues and hospital managers, so this course had the potential to raise research awareness across the institution and to indirectly contribute to postgraduate medical education well beyond those students actually enrolled on the course. There was no mechanism in the local Ghanaian university system for awarding a postgraduate diploma and as the course met the appropriate UK standards [3] it was accredited as a Diploma in Project Design and Management (DPDM) by our UK institution.

Although there is a large literature on different approaches to educational evaluations [11,12] few innovative interventions for work-based education of health professionals in developing countries have been adequately evaluated. The aim of this study was to evaluate the effectiveness of the DPDM course in achieving its learning outcomes and to identify critical elements that promoted learning. We were aware that the evaluation had to be rigorous but also simple and feasible in a resource-constrained setting.

## Methods

### Participants

Study participants were all 15 learners who enrolled on the DPDM course in KATH in 2003 (6) and 2004 (9). None of the learners had had previous experience of designing and implementing their own research and all volunteered to take part in the study. Learners were assured of anonymity and that participation would not affect their academic progress. Learners' specialities included paediatrics, adult medicine, ENT surgery, obstetrics and gynaecology, physiotherapy, pharmacy and health management.

### Selection of evaluation tools

There were no specific evaluation tools available for evaluating such courses in developing countries so we based our evaluation on a published framework incorporating criteria derived from different perspectives [13]. We included indicators of process (i.e. how the course was delivered), content (i.e. what was delivered) and outcomes (i.e. completed assignments and projects; improved competence and confidence in research skills) [14]. The criteria for selecting the evaluation tools were that they had been published in peer-reviewed journals, were relevant to the course outcomes and met requirements for evaluating innovative educational interventions based on social learning [11,12], and they could be applied within the available time and resource constraints. Two different methods were used to assess each of the learning outcomes. Student assessments took place in months 3, 8 and 12; the evaluation was carried out at the end of the course in month 12.

### Evaluation of competence and confidence

Competence was assessed from learners' performance in two assignments, the research proposal (month 4) and project report (month 12). The assignments were marked independently by two markers from either institution or by three markers if marks were discrepant by ≥10%. Four of the authors (one from Liverpool, three from Kumasi) and three other academics who were not involved in this research (one from Liverpool, two from Kumasi), were involved in marking assignments. As part of the examination process, and to ensure that the research did not bias student marks, all marks were reviewed by two independent examiners who had not been involved in the course, one from Liverpool (internal examiner) and one from an external UK university (external examiner). The course curriculum, assessment and final marks were reviewed and agreed by the examiners to ensure fairness and transparency and that the course met UK academic standards [15].

This assessment was complemented by a 10 point Research Self-Efficacy Scale (RSES) which asked learners to score 11 statements about their research skills from 1 (= not at all able) to 10 (= very able) (table 1). The RSES has been used to assess research self-confidence and has good internal consistency and face validity across a range of professional programmes [16,17]. Through group discussions the students identified the key components of the research process in which they were particularly lacking skills and these skill gaps were used to focus the RSES to suit the local context and needs. Through these detailed discussions, students had already begun to learn about research topics and it was felt that completion of the RSES at this stage would not provide a true picture of their baseline self-efficacy in research. Students were therefore only asked to complete the RSES at the end of the course.

**Table 1:** Mean score for each statement on Research Self-Efficacy Scale in order of agreement

**Statement** ***As a result of the course I am able to*.......**	**Mean score (out of 10)**
Identify a clinical problem that is amenable to research	9.70
Produce a realistic budget for my research project	9.00
Formulate a clear research question or testable hypothesis to address a clinical problem	8.71
Write a balanced and comprehensive literature review	8.57
Put together a team to help me to conduct my research	8.50
Teach someone else how to design and implement a simple research project	8.50
Do an effective electronic database search of the literature	8.29
Effectively present my study and its implications	8.29
Choose a research design that will answer my research question or hypothesis	8.21
Design and implement the best data analysis strategy for my research study	7.50
Design and implement the best strategy for collecting my samples	7.42

**Mean score (SD)**	**8.43 (0.63)**

Because the RSES may over-estimate learners' confidence and it may be too insensitive to detect subtle positive perceptions about learners' acquisition of research skills, a 'stages of change' (SOC) tool was used to assess progress in changing learners' attitudes, intentions and actions in relation to research 18]. The SOC tool has been used to describe behaviour change in relation to disseminating research results within a health institution and to highlight where barriers to change may exist. The SOC tool asked learners to state whether they strongly agreed, generally agreed, generally disagreed or strongly disagreed with 13 statements relating to learners' attitudes, intentions and actions towards research (table 2). The total and mean number of learners' responses to each statement was calculated according to the level of agreement of the respondents with the statements; these results were used to derive the total and mean for each category of statements (ie. attitudes, intentions and actions). The number and percentage of individual students who progressed on the SOC model was calculated for each statement and for each category.

**Table 2:** Statements for 'stages of change' model [17] for measuring development of research confidence: learners chose one of four responses for each statement (strongly agree, generally agree, generally disagree or strongly disagree) about their research-related behaviour before and after the DPDM course.

**Attitudes**
Learning research skills is important
Understanding how to do research is relevant to my work
I should incorporate research findings into my clinical practice
I should do more research myself
**Intentions**
I plan to learn more about how to do research
I will bring up the idea of incorporating research into our work with colleagues
I plan to include use of research findings in my clinical practice
I will suggest that we discuss how to improve our use of research results at our departmental meetings
**Actions**
I have suggested casually to some of my colleagues that they should do research
I have spoken in a formal meeting about increasing the amount of research done by our department
I have changed my clinical practice as a result of doing research
I have spoken in a formal meeting (or to my Head of Department) about increasing the use of research/guidelines in our unit
I am currently working on another research project

Corroboration of data obtained from the SOC tool was sought from learners' reflective commentaries concerning their experiences of doing research. The commentaries were analysed by one of the authors (IB) using elements of the grounded theory approach [19]. Codes for analysis of the reflective commentaries in relation to confidence in research skills were derived from data in the first few commentaries. The codes were applied to data that specifically mentioned 'confidence' as well as students' personal, institutional and contextual reasons for lack or acquisition of confidence (e.g. non-clinical status, recognition of application of research to clinical practice). All reflective commentaries were then analysed iteratively until no new themes emerged. Major themes were identified and inferences made about linkages between themes. Reflective commentaries were re-examined for data that may discredit the theories and this information was used to refine and consolidate the theories.

### Identification of factors that promoted learning

The nominal group technique [20] was used to identify elements within the course that most effectively promoted learning. This technique enabled individual learners to contribute to the process equitably and the whole group to identify and rank the critical elements. Using 'post its' each learner wrote three elements about the DPDM course that had facilitated their learning. These elements were pooled, grouped into themes and ranked through learners' group discussions. To corroborate data from the nominal group technique, learners' reflective commentaries were analysed as above but with codes that incorporated skills the students identified they had learnt (e.g. budgeting, time-keeping), the process by which they had learnt these skills (e.g. by doing a pilot study, by reviewing someone else's proposal) and how they would use these skills in their professional work (e.g. improve record keeping, communicate better with laboratory staff).

## Results

Four of the 15 participants were female and the mean time since graduation was 10.3 years (range 2–23 years). All 15 participants completed the research proposal, research report and reflective commentaries. 14 participants completed the RSES and SOC model. 11 took part in the nominal group technique; non-participation was due to clinical or teaching commitments.

### Evaluation of competence and confidence

The course examiners determined that of the 15 participants who completed both the proposal and report course assignments, 15 had passed the project proposal assignment, 14 passed the report assignment (mean mark 61.1 out of 100; SD 8.1) and two gained distinctions (i.e. >70%). The learners' mean (range, SD) score for the 11 statements on the RSES was 8.43 (7.42–9.70, 0.63) out of a possible maximum of 10 (table 1). Overall 82.5% of responses were graded as 8, 9 or 10 (i.e. very able). There were no responses graded <5 (i.e. not able).

Comparison of students' pre- and post-course responses on the SOC model showed that 11 of 14 students improved in research-related attitudes and intentions and 12 improved in actions. The mean number of responses that 'strongly agreed' and 'generally agreed' with the SOC statements pre-course was 2.95 and 7.2 respectively. Post-course these figures improved to 12.8 and 1.9 (table 3). For paired pre- and post-course responses for individual learners, none had regressed on the SOC, and 78%, 81% and 85% had improved by at least one stage in 'attitude', 'intention' and 'action' respectively. (table 4)

**Table 3:** Mean number of responses for statements concerning research-related attitudes, intentions and actions on the stages of change model pre- and post-course

	**Strongly agree**	**Generally agree**	**Generally disagree**	**Strongly disagree**	**Total number of responses**
**Pre-course**					

Attitudes 4 statements	4	6.5	2.75	2.5	63
Intentions 4 statements	3.25	7	3.75	1.75	63
Actions 5 statements	1.6	8	3	3	58

**Overall mean**	**2.95**	**7.2**	**3.2**	**2.4**	

**Post-course**					

Attitudes 4 statements	14.5	1.4	0	0	64
Intentions 4 statements	14.5	1	0	0	60
Actions 5 statements	9.4	3.2	0.2	1	69

**Overall mean**	**12.8**	**1.9**	**0.07**	**0.3**	

**Table 4:** Number of individual students (N = 14) who improved by none, one, two or three levels on the 'stages of change' model post-course compared to pre-course.

**Category**	**Statement**	Number of students who improved by 1, 2 or 3 levels on SOC*				**% of students who improved by ≥1 level**	**Total number of students who responded**
		**0**	**1**	**2**	**3**		

Attitudes	A	4	7	2	1	78%	14
	B	2	9	3	0		14
	C	3	6	3	2		14
	D	3	5	2	2		12

Intentions	E	2	6	3	2	81%	13
	F	2	8	3	1		14
	G	3	8	2	0		13
	H	3	4	2	3		12

Actions	I	3	5	4	1	85%	13
	J	3	6	3	1		13
	K	1	5	3	1		10
	L	1	4	2	3		10
	M	0	3	4	0		7

* Levels improve between strongly disagree, generally disagree, generally agree and strongly agree

Three major themes concerning confidence in research skills emerged from analysis of the learners' reflective commentaries; initial lack of confidence to start research, increased research skills and confidence about undertaking research, and improved self-confidence. Reasons identified by learners' for their initial lack of confidence in starting research were their perception that research was complex and they were disadvantaged because of professional hierarchy and inexperience. Learners' examples of their increased research skills and confidence included their ability to begin new research projects and recognition of the role of research in improving clinical practice. Examples of learners' improved self-confidence were related to acquisition of transferable skills such as the ability to think critically and to contribute to group activities without fear of intimidation (table 5).

**Table 5:** Examples of comments in reflective commentaries regarding confidence in research skills

**Research confidence theme**	**Illustrative extracts from reflective statements**
Reasons for lack of confidence	• *'I did not know how to start. I did not have the confidence to even start something no matter how small it may be'* • *'I felt like I was lost in a maze with an assortment of heavy loads on my back'*
Increased research skills confidence	• *'...going through the course has helped me overcome my fears and anxieties concerning research'* • *'I have acquired skills and confidence needed to design and conduct research projects'* • *'I now feel very confident in discussing matters about research and have developed the interest in converting many clinical problems in the society into clinical research'*
Improved self-confidence	• *'it [the course] has taught me the need to expand my frontiers and be self-confident'* • *'I have enough potential in me to achieve whatever I set my mind to do'* • *'I have had the confidence to pursue anything I intend doing without being intimidated by personalities.'* • *'... [the course activities] have made me a critical thinker'*

### Elements of course that promoted learning

#### Nominal group technique

Participants in the nominal group technique identified 32 elements in the DPDM course that had facilitated learning which were categorised as:

*Course structure*: the work-based, part-time course, delivered through a combination of peer support, short workshops and self-directed learning, motivated learners and optimised their chances of completing the course within time and budget constraints

*Learner-designed course*: learners' ownership of the course design meant that they fully understood what was required to complete the course successfully and they felt empowered to recommend improvements to hospital managers such as better internet access and statistical support

*Reflection about research experiences: *learners recognised that reflective practice was highly effective for promoting understanding of the research process and for improving self-confidence.

#### Reflective commentaries

Analysis of learners' reflective commentaries revealed two major elements that promoted learning (table 6).

**Table 6:** Examples of comments in reflective commentaries regarding elements of the course that promoted learning of research skills

**Factor promoting learning**	**Illustrative extracts from reflective statements**
Learning by doing and reflecting	• *'The experience gained this way, though painful, could be longlasting'*. • *' the road has been turbulent, but perseverance and the will to learn .... have made the journey safe and endurable'*
Learning from peers	• *'This was not an easy time... I must however admit that the committee scrutiny gave me profound understanding into my research topic and infused into me new ideas for writing my proposal'* • *'Sitting in small groups, vetting and contributing to each others work were one of the most useful learning experiences'* • *It [group session] was a good platform for learning and it also made me realise that among my colleagues we had enormous experience and knowledge which when shared and channelled properly could be used for utmost benefit'*

*Learning by doing and reflecting*: in addition to specific research skills that had been learnt by doing research such as piloting research tools, budgeting and statistical methods, learners also acquired generic professional skills such as building and motivating teams, and time management.

*Learning from peers*: despite initially engendering feelings of defensiveness and discomfort, the constructive criticisms and refinement of learners' proposals by the peer review committees and monthly peer support meetings, were highly effective learning mechanisms. Learning from peers improved the quality of research and promoted research-based clinical practice, professional skills including constructive criticism, mutual respect, self-confidence and cross-professional working.

## Discussion

Despite demands for rigorous evaluations of educational interventions and improved knowledge about what makes interventions work there are almost no peer-reviewed published evaluations of UK-accredited courses in developing countries [21,22]. The UK Quality Assurance Agency report only cites two unpublished reviews of courses in China and South Africa, in the last six years [15]. We evaluated the effectiveness of a course to teach research skills to health professionals in Ghana, identified elements that were perceived to be critical in making the course successful and described the evaluation model and its usefulness in a resource-poor setting.

### Principal findings

Using the model we have developed to evaluate a novel educational initiative in a resource-poor setting, we have demonstrated that a learner-designed course to teach research skills to health professionals in Ghana can be effective and meet international standards for quality education. The course met the needs of individual learners, who considered themselves to have become confident and competent in research, and of the institution, which increased its research capacity. A secondary but important outcome was the enhancement of learners' professional skills and better research awareness and advocacy within KATH. Critical to the success of the course was ownership by the learners, support from peers and learning by doing research and reflecting on experiences.

### Strengths and limitations of the study

In developing countries demand for high quality education is increasing. When resources are scarce it is essential to demonstrate that educational interventions are effective. Our study provides a scheme for evaluating an innovative educational course, based on the theory of social learning, which was feasible to implement and interpret in a developing country context. Our study incorporates several features that identify it as research rather than a straightforward evaluation [7,23]. The study used valid, reproducible and appropriate methods leading to neutral conclusions rather than decisions; it was not influenced by the funder and it advanced knowledge, contributed to theory and explored opportunities for transferability. A major strength of this study was the use of diverse methods to examine process, content and outcomes [14] and two different methods [13] to assess the same outputs.

Because of the nature of social learning our results need to be interpreted cautiously because measured benefits may have been influenced by other educational experiences and variations in the students' work or personal environment [7]. In the absence of subject-relevant benchmark statements, assessment of learners' research competence was judged against UK higher education quality standards which included alignment of learning outcomes with curriculum content and assessment, use of assessments to support learning, and opportunities to reflect on learning [3]. Self-reported ratings tend to overestimate student confidence and competence especially when the evaluation is conducted by the tutors as in this study. As skills are learned, students' self-rated ability more closely reflects actual ability levels [24] This 'research-naivety' among the students in our study may partly explain why they had slightly higher post-course scores in the RSES (7.4–9.7) than students in a previous study (55.3 – 82.4, scored out of 100 rather than out of 10: see table II p194 in reference 17). Despite these limitations, the RSES and SOC have been found to be useful for demonstrating the process of developing research competence and confidence and for ranking improvements in research-related attitudes, intentions and actions in previous studies [16-18]. The evaluation methods we used were simple to adapt and use in the Ghanaian setting but their usefulness needs to be assessed in a variety of different developing country contexts. Such tools can benefit from complementary qualitative data from learners' reflections and the nominal group technique to obtain richer and deeper understanding of how learners acquired research skills.

### Transferability of evaluation model to other settings

Each educational setting is unique. Our learners and their learning environment had some characteristics that may not be reproduced elsewhere and which may impact on potential sustainability and transferability of our methods and findings. Our learners were highly motivated to learn research skills in order to pass professional examinations and to obtain an internationally-recognised Diploma qualification. The learners had designed the course themselves and therefore understood what they needed to do to succeed. The experience of future cohorts may be less intense, as they will not be 'pioneers' and this may influence their motivation and commitment to the success of the course. The learners were a unique mix of middle-grade, health professionals who were prepared to share their wealth of pooled expertise to compensate for the lack of local tutors. Learning outcomes can be affected by the social mix, culture and lifestyle of students [25] and our approach may not work so well with more junior or less motivated or cohesive learners. The KATH managers had a clear vision and strategy for developing their institution's research capacity [26] and provided resources rapidly and flexibly to ensure the success of the course. Although by the end of the course only a few students considered themselves to be capable of managing a research project independently, our findings show that through the course the students have developed an appreciation of the process of research and the role that research can play in improving evidence-based clinical care. This will contribute to improving the research culture within the institution [26].

### Study outcomes in the context of theories about social learning

Our research highlights how learners set up their own 'community' of research practitioners at KATH and utilised the learning opportunities provided by the social aspects of their work environment [27,28] to underpin changes at institutional level [5]. Such 'communities of practice' and peer support groups facilitate learning [29], promote reflective interactions [30,31] and generate 'creative turmoil' and innovation [32], and may explain why the DPDM course played such a key role in strengthening KATH's institutional, as well as individuals', research capacity.

To promote knowledge around coherent topics, educational research should be located within a theoretical framework [33]. The course that we evaluated was developed using theories of social learning and workplace-learning synergies. The course evaluation, which demonstrated that students had achieved the learning outcomes, and our research into effective strategies to promote student learning in a resource-poor environment, demonstrated the importance students placed on learning by sharing their own varied knowledge and experiences. Our research therefore confirmed previous findings that learning outcomes are influenced by the social and professional diversity of students [25] as some learners felt disadvantaged within the cohort because of perceived differences in professional status or lack of previous research experience [34].

The findings from our study support theories from developed countries concerning the process of social learning at work [4,8,34]. Key characteristics of work-based learning are that it is managed by the learners, it is team based, innovating and empowering, and it can be enhanced by group activities that promote reflective practice and higher order thinking [35]. Our research has demonstrated that these concepts can be successfully applied to a work-based course for health professionals in Ghana. This study therefore contributes evidence that the process of social learning at work, and theories about the role of social interactions and institutional culture on improving effectiveness of learning which have emanated from developed countries, also apply in developing countries.

### Implications for educators and future research

International development policies are urging UK universities to expand access to high quality education particularly in Africa. There is an urgent need for practical tools, such as rigorous assessment processes [36], RSES, SOC models and qualitative analysis of student reflections, to guide delivery and evaluation of accredited courses in resource-poor countries. These tools should be sensitive to the need to understand and build on social and institutional interactions to promote effective learning. The combination of an innovative learner-designed course underpinned by peer-supported learning, originating in a developing country, and an educational quality framework, generated by developed countries, offered unique opportunities for bilateral exchange of best practice between South and North.

## Conclusion

Quality-assured innovative education programmes for health research can be successful in resource-constrained settings if learners are intimately involved in the design, delivery and assessment of the course and understand the rational and requirements of quality frameworks. It is possible to conduct rigorous evaluations of courses in a resource-constrained setting using a quality framework and published education evaluation tools. Highly motivated learners in a supportive learning environment can be facilitated to pool and share knowledge despite the lack of local tutors and role models. Educational theories emanating from developed countries about promoting effective learning through synergy between the learners' social interactions and their learning environment may be transferable to developing countries

## Competing interests

The author(s) declare that they have no competing interests.

## Authors' contributions

IB conceived of the study and drafted the manuscript

DA contributed to study design, data collection, managing peer-groups, manuscript review and analysis of data

GBA contributed to study design, managing peer-groups and analysis of data

TA contributed to study design and manuscript reviews

ANA contributed to study design and provided institutional support

AYOA contributed to study design, managing peer-groups and data collection

PK contributed to study design, managing peer-groups and analysis of data, and provided institutional support

All authors read and approved the final manuscript

## Pre-publication history

The pre-publication history for this paper can be accessed here:


